# Experimental Induction of State Rumination in Youth Soccer Players on the Pitch: How Can We Evaluate an Effect of Rumination on Soccer‐Specific Performance?

**DOI:** 10.1002/ejsc.70021

**Published:** 2025-08-26

**Authors:** Alena Michel‐Kröhler, Stefan Berti

**Affiliations:** ^1^ Department of Clinical Psychology and Neuropsychology Institute for Psychology Johannes Gutenberg‐University Mainz Mainz Germany; ^2^ Department of Neuropsychology and Psychological Resilience Research Central Institute of Mental Health Mannheim Germany

**Keywords:** ball juggling, competitive sport, experimental sport psychology, unresolved goals

## Abstract

Based on the goal progress theory, we examined whether cueing an unresolved goal triggers state rumination in athletes and consequently leads to performance deterioration. However, experimental approaches are usually conducted in the laboratory and only rarely in the field. Therefore, the aim of this proof‐of‐concept study was to verify whether the findings obtained in previous experimental studies could be transferred and confirmed in the field. For this purpose, the study was applied during regular training sessions. Fifty soccer players (*f* = 17, *m* = 33, age range: 14–18 years; *M*_age_ = 16.38) were divided into three different conditions—one experimental condition in which a goal‐related rumination was induced and two control conditions in which a comparable goal‐related induction or a neutral task was performed. Before and after the inductions during athletic practice, soccer players performed a sport‐specific test to examine potential changes in athletes' performance as a function of condition and answered questions related to their state rumination. Different mixed ANOVAs were performed to examine (1) whether we could significantly elicit rumination in soccer players and (2) whether the application of the experimental procedure had an impact on performance in the subsequent sport‐specific test. Two main findings were identified: First, the experimental procedure generally transfers well to the field. Second, however, the expected effects of state rumination on performance in the sport‐specific test were not obtained. Reasons for the lack of clear effects and approaches for future research are discussed.

## Highlights

- This proof‐of‐concept study transferred an experimental setting from the laboratory into a sport‐specific performance context to test whether rumination (i.e., repetitive intrusive thinking) affects athletic performance.
- We report on an experimental procedure that combines the induction of state rumination with a football‐specific performance test (e.g., ball juggling) in the context of regular training sessions as a research tool in sport and exercise psychology.
- Although the expected effects of state rumination on performance were not obtained so far, we could demonstrate that the experimental procedure is generally well transferable to practice and represents a promising approach that should be further developed in the future to complement correlative studies.

## 1 Introduction

Research in the field of sport and exercise psychology aims to identify psychological factors that are decisive for or prevent the achievement of athletic (peak) performance. A typical approach in this area is to conduct correlative studies. For example, various questionnaires can be used to identify factors that are associated with success (or failure) in sporting competitions. This research approach helps to identify numerous determinants of athletic behavior and develop sophisticated models and theories that coaches and psychologists can use as a basis for their interventions to support athletes. Ideally, however, scientific studies aim to draw causal conclusions. This requires the development and implementation of experimental approaches in sport and exercise psychology. In the current study, we present one such approach. Specifically, we aimed to develop an experimental approach that can be used to test the extent to which recurrent negative thoughts (i.e., rumination) actually influence specific athletic performance. This question is central because the explanation often given for poor athletic performance is that one was mentally blocked by disturbing thoughts. For this reason, sports psychology interventions are often aimed at dealing with performance‐inhibiting thoughts. However, experimental approaches allow clarifying whether negative thoughts actually impair athletic performance and how strong the impact is. Moreover, experimental approaches enable a systematic development and evaluation of specific mental training techniques. We, therefore, developed and applied a paradigm that aimed at testing the influence of induced thoughts on a sport‐specific performance. Briefly, we tested whether rumination impairs the ball juggling performance in young soccer players. To sum up, the outcome of the study is mixed. On the one hand, we demonstrate that it is feasible to apply an experimental approach to athletic performance. On the other hand, the results of the current study do not depict a negative effect of rumination on specific soccer performance. We discuss these results and show how this particular paradigm and experimental paradigms in general can be improved to test causal relationships between mental processes and athletic performance.

Rumination can be defined as a predominantly maladaptive thinking style in which people find themselves in a kind of thought carousel of repetitive intrusive thoughts from which they have difficulty escaping (Watkins and Roberts 2020). Moreover, ruminative thoughts involve a negative perspective and intense analysis of one's own feelings, concerns, and adverse personal experiences (Watkins 2008). Previous studies examining the relationship between rumination and performance have tended to capture performance in the broader sense of goal achievement, career development, and athletic behavior. For example, two studies demonstrated that athletes who achieved their goals at the end of the season reported less rumination compared to athletes who did not achieve their goals (Kröhler and Berti 2017; Michel‐Kröhler et al. 2024). Furthermore, a study with hockey and soccer players showed expertise effects between athletes and nonathletes in terms of reflective rumination (Roy et al. 2016). The authors stated that lower rumination scores were associated with longer careers at a higher level in soccer players. In addition, Scott et al. (2002) reported negative correlations between different partial performances in tennis and rumination. Regarding the relations with athletic behavior, Maxwell (2004) showed significant correlations between anger rumination (i.e., the tendency to obsessively ruminate about experiences), accompanied by negative affect in the form of anger, and self‐reported aggressive behavior in competitive athletes. Finally, a study from Kröhler and Berti (2019) demonstrated that ruminative thinking was related to state orientation, which is the individual's tendency to stick on their own emotions and thoughts while performing under pressure compared to action orientation, which focuses on acting. Taken together, the results of previous studies underscore the association between rumination and various facets of performance in sport. However, the studies were all based on a purely correlational design, which does not allow for causal conclusions.

A promising approach for investigating causal relationships is offered by the goal‐cueing task (Michel‐Kröhler and Berti 2023; Roberts et al. 2013); a procedure that encourages participants to ruminate and examines possible effects on selected outcome variables. The goal‐cueing task is based on Martin and Tesser's Goal Progress Theory (1996, see also control theory by Carver and Scheier 1982, 1990), which states that a perceived discrepancy between the desired goal and the current state can trigger rumination. The goal‐cueing task focuses on unresolved problems and concerns that cause the participant to feel sad, negative, or stressed to trigger rumination in contrast to participants who are dealing with a resolved problem. The goal‐cueing task has been previously studied in various experimental settings in the laboratory in nonathletic contexts (Edwards 2017; Lanning 2015; Michel‐Kröhler et al. 2023; Roberts et al. 2013, 2020) and used in a laboratory study with athletes (Michel‐Kröhler and Berti 2023). Although the above studies all report successful induction of rumination by creating a discrepancy between the current state and the desired goal (i.e., unresolved problems), results are mixed in terms of measured performance (i.e., sustained attention during the Sustained Attention to Response Task; Robertson et al. 1997). Differences between the unresolved and resolved goal condition in sustained attention were found in one study (Roberts et al. 2013), whereas no differences were obtained in four other studies (Edwards 2017; Roberts et al. 2020; Michel‐Kröhler and Berti 2023; Michel‐Kröhler et al. 2023). However, it is unclear to what extent results from pure laboratory studies can be transferred to other, particularly sport‐specific contexts (i.e., performance in training or competitions).

One of the main challenges here is the implementation of experimental designs (i.e., the experimental manipulation of thoughts) in competitive sports or rather during competition. This would prove to be very difficult or not possible (not least because of the potential consequences on the outcome of the competition). Moreover, the causal relationship between rumination and performance (i.e., various forms of attentional performance, problem‐solving behavior, concentration) in and out of the sport context has already been investigated with various laboratory studies (Lyubomirsky et al. 1999; Lyubomirsky et al. 2003; Michel‐Kröhler and Berti 2023; Michel‐Kröhler et al. 2023). However, the applied performance tests in the laboratory do not reflect the complexity of athletic performance, so that a comparison with actual athletic performance is hardly possible. Therefore, a compromise between a performance situation in competition and in the laboratory is needed to adequately and purposefully capture athletic performance. One such compromise is represented by field studies, which can be applied to deepen our understanding of the effects of rumination in real‐world situations.

**FIGURE 1:**
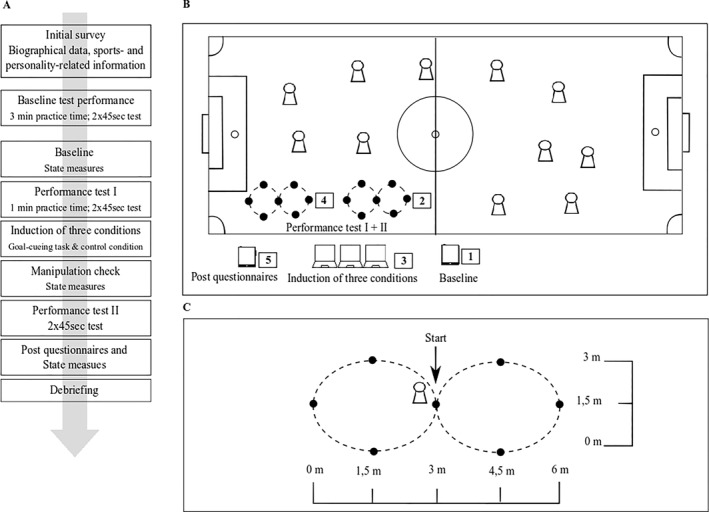
Overview of the setting for the field study. (A) Overview of the procedure starting with the initial survey, in which the trait measures as well as information used for sample description were collected, followed by a detailed explanation and a baseline measurement of the performance test (i.e., ball juggling) in separate appointments. The experimental setting begins with a baseline measurement to assess the athletes' current state of rumination, mood, and affect, followed by the first performance test and subsequent induction of the three conditions to which the athletes were randomly assigned. After the state measurements were queried a second time (as a manipulation check), the second performance test followed. This was followed by the post survey and the third query of the state measures before the study concluded with debriefing. (B) Overview of the detailed experimental setting on the field (i.e., soccer field). The numbers reflect the order of the different test stations according to the procedure of the experimental setting mentioned in (A). (C) Detailed setup of the ball juggling task (i.e., performance test).

The current study aimed to investigate whether induced rumination influences performance in a contextual sport‐specific task by transferring the laboratory design to competitive training situations. Here, we proceeded in two stages: First, we conducted a preexperiment to ensure an optimal implementation of the experimental setting and to design a “best practice model” for our main experiment. Second, to answer our research question, we used an experimental within‐between‐subjects design in our main experiment with two time points (pre‐ vs. post‐induction) and three conditions (experimental [EC] vs. goal‐related control condition [GRCC] vs. neutral control condition [NCC]). Athletes in the EC and the GRCC were subjected to the rumination induction using the goal‐cueing task (Michel‐Kröhler and Berti 2023). In contrast, athletes in the CC were subjected to another control induction, which was not related to personal goals. Before and after the induction, we assessed athletes' current state of rumination, mood, and affect (as manipulation check) as well as their performance in the ball juggling test (task from the test battery of the DFB [“Deutscher Fußball‐Bund e.V.”; Lottermann et al. 2003]). Here, we assume that athletes' performance in the EC deteriorates from the first to the second performance test compared to the other two conditions. The exact description of the performance test and the detailed procedure of the experiment are presented in Figure 1.

## 2 Methods and Materials

### 2.1 Participants

Table 1 summarizes the individual characteristics of athletes separately by condition and for the entire sample. The sample comprised 50 soccer players (17 female, 33 male), who had on average 10.66 (SD = 2.49, range: 5–14 years) years of experience in their sports and were aged between 14 and 18 years (*M =* 16.38, SD = 0.94). They play at the third highest level in Germany and therefore can be regarded as soccer experts in the junior division.

**TABLE 1:** Mean (M), standard deviations (SD), 95% confidence intervals (95% CI), and Cronbach's alpha (*α*) of sample characteristics separated by conditions.

	Experimental condition		Goal‐related control condition		Neutral control condition		Total		
	*M* (SD)	95% CI	*M* (SD)	95% CI	*M* (SD)	95% CI	*M* (SD)	95% CI	*α*
	*n* = 19 (*f* = 7, *m* = 12)		*n* = 16 (*f* = 5, *m* = 11)		*n* = 15 (*f* = 5, *m* = 10)		*n* = 50 (*f* = 17, *m* = 33)		
Sample characteristics									
Age	16.26 (1.04)		16.44 (0.96)		16.47 (0.83)		16.38 (0.94)		
Age range	14–18 years		14–18 years		14–17 years		14–18 years		
Discipline‐specific training/week	4.26 (0.65)		4.06 (0.57)		4.20 (0.41)		4.18 (0.56)		
Additional training/week	2.95 (1.68)		2.25 (1.65)		2.40 (1.68)		2.56 (1.67)		
Hours/week	11.21 (3.72)		9.00 (3.44)		10.00 (3.52)		10.14 (3.62)		
Competitions/year	28.61 (10.36)^a^		33.56 (7.37)		33.07 (15.07)		32.96 (14.75)^a^		
Experience (in years)	10.63 (2.59)		10.62 (2.30)		10.73 (2.71)		10.66 (2.49)		
Baseline stress	27.58 (3.95)	[25.67, 29.48]	24.06 (3.25)	[22.33, 25.80]	25.87 (4.31)	[23.48, 28.25]	25.94 (4.06)	[24.79, 27.09]	0.69
Personality‐related information									
Athletic identity	40.84 (7.85)	[37.06, 44.62]	42.00 (4.59)	[39.55, 44.44]	42.73 (3.22)	[40.95, 44.51]	41.78 (5.71)	[40.15, 43.40]	0.83
Perseverative thinking	30.05 (11.56)	[24.48, 35.62]	24.37 (10.08)	[19.00, 29.75]	26.93 (7.67)	[22.69, 31.18]	27.30 (10.14)	[24.42, 30.18]	0.92
Brooding	10.68 (3.02)	[9.23, 12.14]	9.87 (2.56)	[8.45, 11.28]	10.43 (3.08)	[8.65, 12.21]	10.35 (2.86)	[9.52, 11.18]	0.71
Reflection	9.63 (3.55)	[7.92, 11.34]	8.33 (2.05)	[7.19, 9.47]	9.28 (2.58)	[7.79, 10.78]	9.12 (2.87)	[8.29, 9.96]	0.74
General self‐efficacy	29.26 (2.96)	[27.84, 30.69]	29.93 (3.97)	[27.73, 32.13]	29.93 (4.58)	[27.28, 32.57]	29.67 (3.74)	[28.58, 30.75]	0.80

*Note: f* = female, *m* = male.

^a^ We removed one athlete from the analysis of the number of competitions because he or she reported 100 competitions/year.

Of these, 19 athletes were assigned to the EC, whereas the other 16 athletes performed the task under the GRCC; the remaining 15 athletes were assigned to the NCC. Group assignment was fully randomized without any restriction. In general, athletic identity was very high in our sample, and athletes did not differ across conditions in this regard, *F*(2,47) = 0.47, *p* = 0.63, ƞp^2^ = 0.02 (see Table 1). Furthermore, they also did not significantly differ in their general rumination assessed with the PTQ (Ehring et al. 2011): *F*(2,47) = 1.40, *p* = 0.26, ηp^2^ = 0.06.

### 2.2 Procedure

The study protocol was approved by the local Review Board of the Institute for Psychology at the Johannes Gutenberg‐University Mainz and was conducted according to the guidelines of the Declaration of Helsinki (World Medical Association 2013). Participants were informed about the nature and the procedure of the experiment and gave informed consent before completing the questionnaires. For athletes under the age of 16, written consent from a parent or legal guardian was also required. Participation in this experiment was voluntary and athletes received 12 euros as compensation.

Soccer players from different men's and women's teams from a local soccer club were contacted to participate in the study by their coaches and sporting director. The study consisted of three parts, an initial survey, which were completed online at home, a baseline test performance measurement in the selected sport‐specific test to explain and become familiar with the performance task and to rule out potential practice effects during the experiment, and the experimental setting (see Figure 1A). The latter were carried out during training sessions at the club's local sports facilities.

The initial survey was conducted using SoSci Survey (Leiner 2019) and comprised biographical, sociodemographic questions as well as training‐related information, and different personality questionnaires (see below for detailed descriptions of the utilized questionnaires). The questionnaire data were also the basis for checking whether the randomized groups differed in their trait rumination retrospectively. In detail, athletes were randomly assigned to either the EC, the GRCC, or the NCC. To be able to assume that the athletes in the three conditions fulfill similar initial requirements regarding their characteristic to ruminate, these should not differ significantly in their values of the Perseverative Thinking Questionnaire (PTQ; Ehring et al. 2011).

We measured the performance in the ball juggling test already 1 week before the actual experiment in training to obtain baseline performance and reduce exercise effects during the experiment (for more details on the execution see *Measures*). The athletes received a detailed test explanation and 3 min of practice time in the course before the performance was measured for the first time over 2 × 45 s in ball juggling. The data from this baseline measurement were not used in the later analyses but were used only for familiarization with the performance test. The actual experiment then took place in a separate training session. After a short warm‐up phase, the athletes ran through five different stations of our experimental setting one after the other (maximum three athletes in parallel: see Figure 1B). First, the athletes filled out questionnaire about their current wellbeing (i.e., perceived stress, rumination, mood, and affect). They then performed the first performance test (2 × 45 s), for which they were given 1‐min practice time (but outside the test course) before being subjected to one of three inductions. Here, athletes were randomly assigned to the EC (i.e., unsolved problem condition), the GRCC (i.e., resolved problem condition), or the NCC (i.e., daily routine). All instructions were performed using Inquisit Webplayer 5 (Free Inquisit 5 Player app, Millisecond Software, Seattle, WA, n.d.). Subsequently, athlete wellbeing was queried again as a manipulation check, more specifically to examine whether induction was successful with respect to state rumination. Immediately afterward, the athletes performed the second performance test (2 × 45 s) before undergoing a final post survey on the tablet. Finally, the debriefing took place, in which the athletes renewed their consent after being fully informed.

### 2.3 Measures

We summarized details of the applied trait questionnaires that we used for sample description in Supporting Information S1. In Table 1, mean values, standard deviations, 95% confidence intervals as well as Cronbach's alpha (*α*) of the respective scales are presented. Unless otherwise stated, we report the sum score of the scale used.

#### 2.3.1 State Measures During the Experimental Setting

*Perceived stress*. We used the Perceived Stress Scale (PSS‐10, Schneider et al. 2020 English original by Cohen et al. 1983) to assess the degree of how participants perceive situations in their lives as uncontrollable, unpredictable, and overloaded relative to their subjective coping abilities. The PSS‐10 comprises 10 items (e.g., “In the last month, how often have you felt nervous and stressed?”) and is answered on a 5‐point Likert scale ranging from “1” (*never*) to “5” (*very often*). Here, we report the general PSS‐10 score. Cronbach's *α* for the entire PSS‐10 is 0.88 in the original study (Schneider et al. 2020).

*State Rumination*. We tested state rumination with three measures: First, we assessed the momentary ruminative self‐focus (Moberly and Watkins 2010). For this purpose, two items (“How much did you focus on your feelings?”; “How much did you focus on your problems?”) are combined into one index and rated on an 11‐point scale ranging from “0” (*not at all*) to “10” (*very*). We refer to this measure as the *ruminative self‐focus* and reported the mean of the two items. Second, we used a single item (“To which extent did you ruminate over something?” see also Koval et al. 2015) to assess general state rumination, which was also rated on the same 11‐point scale. We refer to this measure as the *general state rumination rating*. Third, we applied a measure to capture momentary repetitive negative thinking (MRNT, i.e., process‐related scale; Rosenkranz et al. 2020). The scale comprises four items (e.g., “Thoughts come to my mind without me wanting them to”.), each of which focused on a core characteristic of rumination—repetitiveness, intrusiveness, uncontrollability, and impairment. Participants answer each statement on a 7‐point scale ranging from “1” (*not at all*) to “7” (*very*). The mean value of the four items is used for the analyses.

*Mood*. We assessed the Multidimensional Mood Questionnaire (MDMQ; Wilhelm and Schoebi 2007) to measure three basic dimensions of mood—valence, energetic arousal, and calmness. The MDMQ consists of six items and is a bipolar measure that comprises three pairs of adjectives rated on a 7‐point scale describing opposite end points of different mood dimensions (e.g., energetic arousal: tired vs. awake, full of energy vs. without energy).

*Affect*. We used the International Positive and Negative Affect Schedule Short Form (I‐PANAS‐SF; Thompson 2007; English original version by Watson et al. 1988) to measure the affective state. The I‐PANAS‐SF contains 5 items each for measuring positive and negative affectivity (PA and NA). Participants were asked to rate the degree to which they feel the emotional state described in each item, on a five‐point Likert scale, ranging from “1” (*very slightly or not at all*) to “5” (*extremely*).

#### 2.3.2 Goal‐Cueing Task

To induce rumination in the EC group, we used a modified version of the goal‐cueing task by Michel‐Kröhler et al. (2023; original version by Roberts et al. 2013). This protocol consists of three successive steps. First, athletes in the EC were instructed to identify an ongoing unresolved goal that repeatedly troubled them, causing them to feel sad, negative, or stressed during the previous week. Athletes were then asked to outline their problem in 5 to 10 sentences (Step 1—goal identification). Second is the evaluation of certain characteristics of the identified goals by answering six different questions (Step 2—goal evaluation). They were asked to what extent the unresolved goal had been bothering them at its worst and at the time of the experiment. Furthermore, participants indicate how important the goal is, how much the problem in the individual goal achievement process exemplifies more general problems, how long the problem exists, and how much time they spend thinking about the problem last week. The questions were each answered on an 11‐point scale from “0” to “10.” Furthermore, these can be used alongside the state rumination measures as a manipulation check for the goal‐cueing task. The third step consists of a 5‐min goal focus phase (Step 3—goal focus period) that encourages athletes to reflect on their identified problem. Example instructions are “Think about what is important about this difficulty in terms of your personal goals,” or “Focus on the aspects of the difficulty that repeatedly come to mind”. Athletes in the GRCC followed the same protocol with one central change: In GRCC, the athletes were instructed to identify a problem from the past that has since been resolved, has not come to mind in the past, and no longer makes them feel bad, sad, down, or stressed. Like in the EC, participants were asked to briefly outline the identified problem in writing, answer some questions about it, and follow a 5‐min audio instruction (e.g.,: “Think about why solving this problem will make progress toward your personal goals.”; for detailed information see Roberts et al. 2013).

In contrast with the EC and GRCC, athletes in the NCC were given a neutral writing task that did not relate to a personal goal but had the same extent. Athletes were asked to describe what they did from the morning until the time of the experiment (see Konig et al. 2014; Yu‐Hsin Liao et al. 2012) instead of identifying a problem of their individual goal achievement process.

#### 2.3.3 Performance Task and Equipment

We measured sport‐specific performance with ball juggling ability, which is a task from the test battery of the DFB talent development program (Lottermann et al. 2003). The test consists of juggling a ball, strictly alternating the left and the right foot, from the starting position and completing as many partial distances as possible within 45 s. The players run through the course in the shape of a figure eight (standardized dimensions see Figure 1C). Each completed part of the course (marked with a cone; see Figure 1C) is scored with one point. Each player has two trials. Both test results are entered as point values in the test protocol. A test ends after 45 s or if the test conditions are not met. These are (a) the start of the play is done from the hand, (b) juggling is done exclusively with the feet alternately, (c) the marker plates must be passed by the player on their outer sides, and (d) the time limit is 45 s (the trial will be aborted at the latest and the distance covered until then is counted). Before the start of the first performance test, the athletes had 1 min to warm up with the ball (but not in the course). Athletes used a full‐sized German football league (DFL) standard football (size 5). The marking cones used to set the two figure eights had the following format: 17 × 14 × 16.98 cm; 510 g. To time the preparation and the 2 × 45 s test time per athlete, stopwatches were used (Model: Schütt stopwatch PC‐90), as well as whistles to signal the beginning and end or, if necessary, the termination of the test time.

#### 2.3.4 Relevance of Performance Task

We captured the relevance of the performance task with two questions: (1) “How important is it for you to do well on the performance test?” and (2) “How important is it for you to do well on the performance test, given your role on the team?”. Both questions were rated on a 10‐point scale ranging from “0” (*not at all important*) to “10” (*fully important*). In addition, we asked athletes whether they had trained for ball juggling in the time between the baseline performance tests and the execution of the study. The response format was “yes” “no” or “I have not yet participated in the baseline measurement at the time of the survey.”

#### 2.3.5 Post Evaluation of the Identified Problem

After completing the experimental part, athletes in the EC and GRCC indicated (1) to what extent their focus was mainly on negative aspects, (2) on bad feelings, (3) to what extent thinking about the problem made it seem worse, and (4) made them feel worse (questions were adapted from Mosewich et al. 2013). Participants rated all questions on a 5‐point scale ranging from “1” (*not at all*) to “5” (*very*).

#### 2.3.6 Statistical Analyses

Collection of experimental data was carried out with the Inqusit Webplayer (Free Inquisit 5 Player app, Millisecond Software, Seattle, WA, n.d.) and data preparation and all statistical analyses were performed with the software R Studio (R Core Team 2018).

To analyze the effects of the goal‐cueing task on state rumination, mood, and affect, we applied single mixed analyses of variance (ANOVAs) using the ezANOVA‐function (“ez”‐package; Lawrence 2016) with time (before goal‐cueing task, after goal‐cueing task, and after the experiment) and condition (EC, GRCC, and NCC) as a factor. Beforehand, we checked the requirements for the application (normal distribution and homogeneity of variances). We conducted a Shapiro–Wilk test for testing the assumption of normality (*p* > 0.05) and a Levene's test for testing the homogeneity of variance (*p* > 0.05).

To compare the athletes' performances in the different conditions, we applied single mixed ANOVAs with time (first performance test and second performance test) and condition (EC, GRCC, and NCC) as a factor. We calculated the analysis with the higher value achieved in each of the two trials per performance test (i.e., maximum score), with the sum value from both trials and with the value from the first trial of each performance test. This results in three different ANOVAs to capture athlete performance. However, typically, the maximum score is used, which is why we focus on this in the main analysis and report the results of the other performance analyses as an addition in the Supporting Information S1. Beforehand, we checked the requirements for the normal distribution of the data and the homogeneity of the variances mentioned earlier.

Regarding participants' characterization of problems in the individual goal achievement process and their evaluation of the identified problem after the experimental setting, we analyzed mean differences between conditions with independent *t*‐tests. In case of nonparametric distribution, we reported the significance of the Wilcoxon signed‐rank test as a robust alternative for independent *t*‐tests (*p*_wilcox_; Field et al. 2012).

## 3 Results

### 3.1 Manipulation Check

The results of the manipulation check in relation to perceived stress as well as athletes' ratings of goal characteristics and goal evaluation can be found in the supplement (see Supporting Information S1). In the following, only the inferential statistics for all state variables used in the manipulation check are reported. Table 2 summarizes mean values, standard deviations, and Cronbach's alpha coefficients (where applicable) for these separated by condition and time.

**TABLE 2:** Mean values (M), standard deviations (SD), and Cronbach's alpha (*α*) of relevant experimental state variables separated by condition and time.

		Experimental condition			Goal‐related control condition			Neutral control condition		
		Time 1	Time 2	Time 3	Time 1	Time 2	Time 3	Time 1	Time 2	Time 3
	*α*	*M* (SD)	*M* (SD)	*M* (SD)	*M* (SD)	*M* (SD)	*M* (SD)	*M* (SD)	*M* (SD)	*M* (SD)
State rumination										
MRNT	0.89	3.33 (1.60)	3.89 (1.64)	3.78 (1.65)	3.00 (1.05)	3.03 (1.16)	2.69 (0.94)	3.18 (1.46)	3.18 (1.51)	2.82 (1.16)
Ruminative self‐focus		3.81 (1.28)	5.05 (1.06)	4.16 (1.37)	4.31 (1.36)	5.12 (0.76)	4.43 (1.32)	4.03 (1.65)	4.43 (1.51)	3.37 (1.63)
General rumination rating		3.58 (1.50)	4.52 (1.35)	3.89 (1.59)	3.12 (1.09)	3.56 (1.31)	3.25 (1.18)	3.40 (1.59)	3.67 (1.34)	2.87 (1.64)
Affect										
Positive affect	0.67	17.05 (2.76)	16.16 (2.52)	16.89 (2.79)	17.12 (3.77)	16.00 (2.63)	16.00 (2.80)	18.40 (5.83)	17.53 (2.97)	17.53 (3.29)
Negative affect	0.79	10.05 (3.27)	9.63 (4.61)	9.58 (3.83)	6.75 (2.98)	7.62 (3.12)	7.56 (3.32)	8.13 (2.72)	6.87 (1.68)	7.20 (2.21)
Mood										
Energetic arousal		3.03 (0.82)	3.71 (1.38)	3.13 (0.70)	3.03 (0.83)	3.44 (1.21)	3.06 (0.83)	2.93 (0.46)	3.67 (1.23)	3.10 (0.43)
Valence		3.47 (0.82)	3.16 (1.07)	2.97 (0.59)	3.41 (1.08)	3.50 (1.14)	3.12 (0.46)	3.43 (0.53)	3.80 (0.94)	3.43 (0.56)
Calmness		3.18 (0.75)	3.47 (1.18)	2.97 (0.39)	2.84 (0.99)	3.94 (1.03)	3.03 (0.67)	3.13 (0.85)	3.83 (0.82)	3.07 (0.84)
Perceived strain		3.10 (1.49)	4.63 (1.16)	3.79 (1.44)	2.87 (1.45)	2.75 (1.29)	2.75 (1.24)	3.00 (1.36)	3.27 (1.28)	2.73 (1.33)

*Note:* Time 1 = before the goal‐cueing task, Time 2 = after the goal‐cueing task, Time 3 = after the experimental setting. MRNT = Momentary repetitive negative thinking (*n* = 49), Cronbach's alpha for the present sample was calculated from the data of the first time point (Time 1, preinduction).

### 3.2 State Rumination

There was a significant main effect of time on ruminative self‐focus, *F*(2,94) = 14.53, *p* < 0.001, *ηp* ^2^ = 0.24, indicating an increase in athletes ruminative self‐focus from pre‐ to post‐induction and a slight decrease from post‐induction to the end of the experiment. In addition, there were no significant main effects of condition, *F*(2,47) = 1.34, *p* = 0.27, *ηp* ^2^ = 0.05, and the interaction *F*(4,94) = 1.48, *p* = 0.21, *ηp* ^2^ = 0.06. The same pattern of results was observed for the general rumination rating; the main effect of time was significant, *F*(2,94) = 5.50, *p* < 0.01, *ηp* ^2^ = 0.10, indicating the same temporal progression as the ruminative self‐focus. In addition, the group effect, *F*(2,47) = 2.05, *p* = 0.21, *ηp* ^2^ = 0.08, and the interaction effect, *F*(4,94) = 1.10, *p* = 0.36, *ηp* ^2^ = 0.04, were both not significant. Regarding the MRNT, results also showed a significant main effect for time, *F*(2,92) = 3.63, *p* = 0.03, *ηp* ^2^ = 0.07, in the already mentioned direction, and a nonsignificant main effect for condition, *F*(2,46) = 1.97, *p* = 0.15, *ηp* ^2^ = 0.08. However, there was a trend toward significance with respect to the interaction, *F*(4,92) = 2.27, *p* = 0.06, *ηp* ^2^ = 0.09. Descriptive statistics showed that the MRNT scores in GRCC and the NCC stayed nearly stable from pre‐ to post‐induction and decreased under the baseline level at the end of the experiment. Athletes in the EC showed a slight increase in their MRNT scores from pre‐ to post‐induction with only a slight decrease to the end of the experiment.

### 3.3 Perceived Strain

There was a significant main effect of condition on athletes perceived strain, *F*(2,47) = 4.78, *p* = 0.01, *ηp* ^2^ = 0.17, and a significant main effect of time, *F*(2,94) = 4.39, *p* = 0.01, ηp^2^ = 0.08, which were qualified by a significant interaction between time and condition, *F*(4,94) = 3.50, *p* = 0.01, *ηp* ^2^ = 0.13. Pairwise comparisons revealed a significant increase in athletes perceived strain from Time 1 (before goal‐cueing task) to Time 2 (after goal‐cueing task) in the EC (*p*_bonf_ = 0.01), and no significant changes in the GRCC and the NCC (both *p*_bonf_ > 0.99). The conditions did not differ in their perceived strain prior to the goal‐cueing task, but significantly differed after the goal‐cueing task, reflecting higher values for athletes in the ECs compared to athletes in the GRCC (*p*_bonf_ < 0.01). There was no significant difference between the EC and the NCC after the goal‐cueing task (*p*_bonf_ = 0.14). With regard to the third time point, descriptive findings indicated a decrease in the EC and NCC, whereas perceived strain in the GRCC stays on the post‐induction level.

### 3.4 Positive and Negative Affect

There was a significant main effect of condition on the negative affect, *F*(2,47) = 4.55, *p* = 0.01, ηp^2^ = 0.16, indicating a higher negative affect of athletes in the EC compared to the GRCC and NCC. In addition, there was no significant main effect of time, *F*(1,47) < 1, or the interaction, *F*(2,47) = 1.39, *p*[GG] = 0.35, *ηp* ^2^ = 0.06. Regarding the positive affect, results showed a significant main effect of time, *F*(2,94) = 4.96, *p*[GG] = 0.01, ηp^2^ = 0.09, indicating that athletes’ positive affect decreased as time progressed. In addition, there were no significant main effects for condition, *F*(2,47) = 1.32, *p* = 0.27, *ηp* ^2^ = 0.05, or the interaction, *F*(2,47) < 1.

### 3.5 Mood

The results of three additional 3 × 2 ANOVAs for the three mood dimensions showed no significant main effects related to condition (energetic arousal and calmness: *F*'s[2,47] < 1, valence: *F*[2,47] = 1.99, *p* = 0.15, *ηp* ^2^ = 0.08) or related to the interaction (all *F's*[4,94]). In addition, main time effects were significant for athletes' energetic arousal, *F*(2,94) = 7.38, *p*[GG] < 0.01, *ηp* ^2^ = 0.13, and their calmness, *F*(2,94) = 10.65, *p* < 0.001, *ηp* ^2^ = 0.18, indicating an increase in these mood dimensions from pre‐ to post‐induction and a slight decrease from post‐induction to the end of the experiment. This did not apply for valence, *F*(2,94) = 2.04, *p* = 0.13, *ηp* ^2^ = 0.04.

To summarize, the 3 (condition: EC, GRCC, NCC) × 3 (time: before goal‐cueing task, after goal‐cueing task, at the end of the experiment) mixed ANOVAs showed a significant increase in the athletes' state rumination from before to after the goal‐cueing task in all three measures. However, the expected interaction effects obtained only a trend toward significance for the momentary repetitive negative thinking index, indicating a potential effect of the manipulation. Regarding the further time course after the experimental manipulation until the end of the experiment, a decrease of ruminative self‐focus and the general rumination rating was observed regardless of the condition, even falling below the baseline level. This did not apply to the MRNT, which remained at virtually the same level.

### 3.6 Performance in the Ball Juggling Test

Athletes indicated at the beginning of the experimental setting that ball juggling had a mean relevance of 5.92 (SD = 2.01) for them. The relevance was rated slightly higher when the athletes considered their role in the team (*M* = 6.26, SD = 2.34). Athletes did not differ significantly in terms of their rating of relevance in the conditions (mean relevance: *F*[2,47] < 1; relevance taking into account one's own role in the team: *F*[2,47] = 1.49, *p* = 0.24, *ηp* ^2^ = 0.06). In addition, 10 athletes (EC = 5, GRCC = 2, NCC = 3) reported training for ball juggling before the study was conducted, whereas 34 did not. Four athletes had not yet participated in the baseline performance measurement at the time of the survey.

Supporting Information S1: Table S2 shows means and standard deviations of the scores obtained for the first and second trials of the performance test as well as the means and standard deviations for the maximum score of the better trial and the sum score of both trials.

**FIGURE 2:**
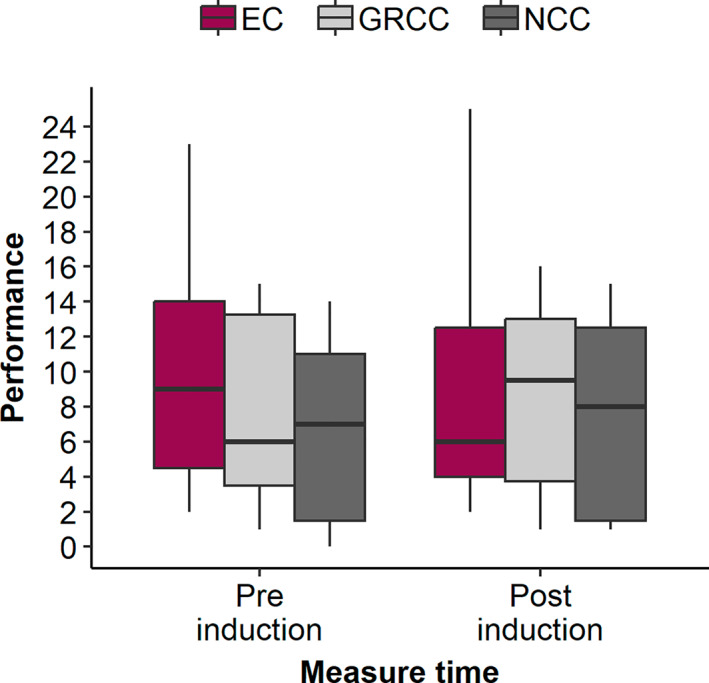
Boxplot showing the distribution of the maximum scores in the ball juggling test separated by time (pre‐ vs. post‐induction) and condition. The boxes represent the interquartile range (IQR), with the horizontal line indicating the median. Whiskers extend to 1.5 times the IQR. EC = experimental condition (i.e., rumination induction), GRCC (i.e., goal‐related control condition), NCC (i.e., neutral control condition).

Results of the main analyses, a 3 (condition: EC, GRCC, NCC) x 2 (time: performance test 1, performance test 2) mixed ANOVA, showed no significant main effects for the maximum score (main effects time and condition: both *F*'s < 1, interaction: *F*[2,47] = 2.06, *p* = 0.14, *ηp* ^2^ = 0.08). However, descriptive statistics indicated a decreasing trend in performance in the EC from pre‐ to post‐induction, whereas performance in the other two conditions tended to increase (see also Figure 2).

## 4 Discussion

The present study aimed to transfer the goal‐cueing task from the laboratory into a sport performance context environment to examine whether rumination affects the athletic performance as mirrored in a sport‐specific task (i.e., ball juggling). To do this, athletes were assigned to one of three conditions in which they identified either an unresolved problem, or an already resolved problem in their individual goal achievement process, or worked on a task that was not related to personal goals. We assessed different measures of state rumination, mood, and affect before and after the goal‐cueing task, which served as a manipulation check for a successful induction. To detect a change in performance because of induced state rumination, athletes performed a ball juggling test immediately before and after the goal‐cueing task. Here, we assumed that athletes' performance in the EC deteriorates from the first to the second performance test compared to the other two conditions. To make a long story short, our results did not show any effect of rumination on athletic performance in this setting. However, in the following, we will discuss possible problems and potential suggestions for improvement in future studies. We will discuss this from a content perspective followed by a formal, conceptual perspective.

### 4.1 Evaluation of the Experimental Setting and Results From a Content Perspective

Our intention was to examine the relationship between rumination and performance in an experimental setting that was transferred from the laboratory to the field. However, our study did not obtain the expected differences between conditions in state rumination after induction, as has been confirmed several times in laboratory studies (e.g., Michel‐Kröhler and Berti 2023; Michel‐Kröhler et al. 2023). On the one hand, this could be due to the lack of variation in the mindset in the sample (i.e., same club, same training philosophy, same performance level, and similar experience in the sport). On the other hand, this could also be related to the fact that athletes in the GRCC and in the NCC also reported thinking about a current problem while conducting the study. Because an individual pursues many different goals simultaneously, reflection on an achieved goal can trigger the processing of currently pursued unachieved goals (Shah 2005). Furthermore, information associated with unachieved goals then has higher availability, is prioritized accordingly, and thus automatically attracts attention (Rothermund 2003). However, we have no information about when and how long or how intensively they thought about a current problem during the experiment, so that the information may need to be put into perspective. Another explanation could be that—specifically in competition athletes—rumination has an adaptive effect and was thus more likely to contribute to problem solving (Watkins and Roberts 2020). In contrast, some athletes from the EC stated that they had not thought of any problem during the study implementation, which could also have affected the ratings of state rumination. However, this is surprising considering the aspect that all athletes in the EC defined and evaluated a problem. Thus, three aspects can be used to explain this fact: (1) Athletes, especially in the EC, might be afraid to show “weakness” by telling the truth, or they might assume that they are expected to focus exclusively on the task at hand and block out all other thoughts. (2) Due to their already long experience in sports, the athletes might have already consciously or unconsciously acquired regulation strategies in dealing with disturbing, negative thoughts, which, for example, became effective immediately after induction. (3) Ball juggling could also be seen as a welcome distraction to switch from a self‐referential focus to an external focus. This would fit with the findings from the clinical and nonsporting context that physical activity can reduce rumination to varying degrees of effectiveness (see e.g., Wang et al. 2025). Accordingly, even a brief sporting activity may be sufficient to divert an athlete's ruminative focus in a sports context. However, this is only speculation at this point. Future studies should systematically investigate whether brief sporting tasks consistently lead to a reduction in rumination. In addition, future studies should include questions about social desirability and knowledge and use of different coping strategies to better embed the results in the overall context.

However, the indirect measures, such as the responses to the goal characteristics and the goal evaluation at the end of the experiment, confirm the expected differences between the EC and the GRCC, which are thus indicative of a successful, albeit weaker, induction. Here, for example, the athletes in the EC indicated that the identified problem bothered them more than the athletes in the GRCC at the time of the experiment and that the problem seemed worse to them at the end of the study and the focus was more on negative feelings. This is also supported by the results regarding perceived stress and negative affect: Athletes in the EC showed significantly higher values than the GRCC, which can be interpreted as a consequence of preoccupation with the unsolved problem. The question of why the results is not mirrored in the state rumination measures remained open.

With respect to performance in the ball juggling test, we were unable to confirm our hypothesis that athletes in the EC showed a deterioration from the first to the second performance test. The descriptive statistics show an approximation of achieved scores in the ball juggling in the sense that the scores obtained in the EC decrease from the first to the second performance task, whereas the scores obtained in the GRCC and the NCC remain largely stable or improve. However, the differences were not significant and the pattern was also retained, even if the performance was calculated using the sum score instead of the maximum score (see Supporting Information S1). The lack of differences in performance data could also be due to the lack of variations in the sample. In addition to the aspects of the sample just mentioned, it has already been shown in previous studies that athletes who have advanced to the elite level are more technically competent (Höner et al. 2017; Bergkamp et al. 2019) and have better perceptual‐cognitive abilities compared to less experienced athletes (Ward et al. 2013; O'Connor et al. 2016). The present sample is a quite homogeneous group of high‐performing youth players at the third highest league, so there is hardly any variation in terms of technical ability. This is accompanied by the fact that ball juggling is proven to be a significant predictor of talent only in the younger age groups in previous studies (Höner et al. 2021), which is why this test is only regularly applied in the DFB talent development program up to the U15 level. Accordingly, other skills (e.g., position‐specific tests closer to competition) may be more important for older age groups. The consideration of testing other skills fits also to findings of Altamirano et al. (2010), who stated that the cognitive processing of ruminators may inherently be geared more toward goal maintenance (i.e., ball juggling) than toward goal shifting (i.e., position‐specific tests). However, Altamirano et al. (2010) refer to depressed ruminators with trait‐like rumination characteristics. It is therefore unclear to what extent this finding can also be transferred to short‐term induced rumination.

### 4.2 Evaluation of the Experimental Setting and Results From a Formal, Conceptual Perspective

Transferring the experimental setting from the laboratory to a real‐world environment was successful in principle, if considered only as a proof‐of‐concept study. Our focus was on an efficient and time‐saving implementation of the experimental setting, which could be easily integrated into the daily training, meant a low workload for coaches and athletes, and increased their willingness to participate. Therefore, among other things, we selected the ball juggling test to capture the sport‐specific performance in athletes, which was easy and time‐saving to integrate into ongoing training with its total of 4 × 45 s (plus additional 1 min preparation time). However, what turned out to be an advantage in advance might now have turned out to be a disadvantage in retrospect. A potential problem in the selection of the test could be that the importance of the performance situation might not have been high enough to be considered an approximation of the pressure in competition (Baumeister and Showers 1986). This would also correspond to the athletes' ratings of the task's relevance, which was rated as medium on average. Here, for example, more complex tests that are closer to the dynamic game situations in soccer would be more suitable for future studies. Another problem could be the variability of the performance in the ball juggling test. This may also be due to the age and level of the sample (see also argumentation in from a content perspective). Therefore, if the ball juggling test continues to be the test of choice in future studies, then it is recommended that multiple ball juggling trials be conducted to provide more information about the stability of the test to obtain more meaningful results regarding athlete performance. Another advantage, which in retrospect could prove to be a disadvantage, is integrating the experiment into ongoing training activities, due to the parallel training of sometimes up to three different teams on one soccer field or interactions between athletes (e.g., observation or commenting on the athletes during the execution of ball juggling; questions to the experimenters or athletes from other athletes who were just in the vicinity, presence of the coach, etc.). This made it hardly possible to achieve the isolation of the athletes and control of confounding variables in order to obtain significant results. However, the question arises whether the interaction of the athletes in the sense of a “systematic group setting” can be used for future studies according to the motto: To what extent is it enough for teammates to observe an athlete, comment on his or her performance, and thereby trigger certain thought processes in him or her? Accordingly, future field studies should consider different applications of induction and, for example, investigating the effectiveness of an isolated individual setting (“How does the individual athlete respond to rumination induction?”) or a proposed group setting.

Finally, as has been shown in many field studies (e.g., Geukes et al. 2017; Mesagno et al. 2020) before, clear hypothesis testing is hardly possible or realistic, as it is difficult to come up with an appropriate sample size, especially when the performance level plays a role. Our findings of this field study were based on a highly selective, albeit relatively small sample (*N* = 50), and as such have left the study statistically underpowered. Post hoc power analyses indicated that with a sample size of 50, the power was 0.52 for detecting an effect size of *ηp* ^2^ = 0.08 (*f* = 0.29) with an alpha level of *α* = 0.05 for the ANOVA with the maximum scores in ball juggling. Thus, to increase the power of field studies with small sample sizes in the future, Simmons et al. (2011) recommend ensuring that there are at least 20 participants in a group.

## 5 Conclusion

The purpose of this proof‐of‐concept study was to transfer an experimental setting from the laboratory into a sport‐specific performance context to test whether rumination affects athletic performance. On the one hand, we cannot proof that rumination does affect athletic performance in our study. On the other hand, the study demonstrates that experimental testing of this hypothesis is feasible. In general, we demonstrate that it is possible to narrow the gap between observing athletic performance (typically constituted in sports competition) and laboratory research by applying the experimental setting into sports practice context. This is a promising approach that should be developed further because it complements correlative studies. A combined research strategy in sport and exercise psychology enables a deeper understanding of psychological and mental factors enabling or limiting peak athletic performance.

## Ethics Statement

The study protocol was approved by the local Review Board of the Institute for Psychology at the Johannes Gutenberg‐University Mainz and was conducted according to the guidelines of the Declaration of Helsinki (World Medical Association 2013). Participants were informed about the nature and the procedure of the experiment and gave informed consent before completing the questionnaires. For athletes under the age of 16, written consent from a parent or legal guardian was also required. This information is also part of the manuscript and is described in detail in the “*Procedures*” section. Affiliation is anonymized for the review process.

## Conflicts of Interest

The authors declare no conflicts of interest.

## Supporting information

Supporting Information S1

## Data Availability

The data supporting the findings of this study are openly available at OSF: https://osf.io/exjf9/.
