# Supplementary material for: Experimental Induction of State Rumination in Youth Soccer Players on the Pitch: How Can We Evaluate an Effect of Rumination on Soccer‐Specific Performance?
Source: Eur J Sport Sci. 2025 Aug 26;25(9):e70021. doi: 10.1002/ejsc.70021 (PMC12380194; doi:10.1002/ejsc.70021)
Supplement: Supplementary file 1 — Supporting Information S1 [file EJSC-25-e70021-s001.docx]

**Supplement S1**

**Measures**

In the following we describe the applied trait questionnaires that we used for sample description (the German versions of the questionnaires were used).

*Trait measures*

*Perseverative Thinking.* The Perseverative Thinking Questionnaire (PTQ; Ehring et al., 2011) is a content-independent self-report questionnaire of repetitive negative thoughts. The PTQ consists of 15 items (e.g., “Thoughts come to my mind without me wanting them to”) and is rated on a 5-point scale, ranging from ‘0’ (*never*) to ‘4’ (*almost always*). Here, we report the general PTQ score.

*Brooding and Reflection.* Huffziger and Kühner (2012) validated the 10-item short version of the Response Styles Questionnaire (RSQ; original English version: Treynor et al., 2003; long version: Nolen-Hoeksema, 1991) with the facets Reflection and Brooding. In the RSQ, it is assumed that brooding describes dysfunctional ruminating about an unattained goal (e.g., “What am I doing to deserve this?”), while reflection describes a more goal- and solution-oriented self-reflection (e.g., “I write down what I am thinking and analyze it.”). Each scale comprises five items. Participants rated all 10 items on a 4-point Likert Scale ranging from ‘1’ (*almost never*) to ‘4’ (*almost always*).

*Athletic identity.* We measured athletic identity using the Athletic Identity Measurement Scale (AIMS-D; Schmid & Seiler, 2003; English original by Brewer et al., 1993), which consists of seven items and is answered on a seven-point scale ranging from ‘1’ (*does not apply at all*) to ‘7’ (*fully applies*).

*Self-efficacy.* We used the General Self-Efficacy Scale (GSE; Hinz et al., 2006 English version: Schwarzer & Jerusalem, 1995) to assess participants’ general sense of perceived self-efficacy, for instance in relation to coping with everyday life or after experiencing all kinds of stressful life events. The GSE comprises 10 items (e.g., “I can always manage to solve difficult problems if I try hard enough.”) and is answered on a 4-point scale ranging from ‘1’ (*not at all true*) to ‘4’ (*exactly true*).

**Results**

***Manipulation check***

Before the experiment started, we measured athletes’ perceived stress with the PSS-10 (Schneider et al., 2020) to ensure that athletes in the three conditions participated in the experiment with the same baseline levels (see Table 1). Results of a one-way ANOVA indicated that there were a significant difference between the conditions, *F*(2,47) = 3.61, *p* = .03, ηp² = .13. A post-hoc analysis showed that athletes in the EC showed significantly higher values on the PSS-10 compared to the GRCC (*p*_bonf_ = .03, see Table 1).

Table S1 summarizes the athletes' ratings of goal characteristics and goal evaluation and the results of the respective test statistics. Regarding the goal characteristics, a *t*-test confirmed that athletes in the EC significantly differ from the GRCC in the extent to which the problem had bothered them at the time of the experiment. Athletes in the EC reported that the problem bothers them more at the time of experiment compared to the GRCC. In contrast, there were no differences regarding the extent to which the problem had bothered them at its worst, the amount of time they spent on the problem, in the evaluation of the goal importance, the duration of the problem, and the extent to which the goal achievement process exemplifies more general problems.

**Table S1**

*Mean (M), standard deviations (SD), test statistics, and effect sizes of the goal characteristics for both experimental conditions.*

|  | **EC** | |  | **GRCC** | |  | ***t*-statistic** |
| --- | --- | --- | --- | --- | --- | --- | --- |
| ***Goal characteristics**** | ***M*** | ***SD*** |  | ***M*** | ***SD*** |  |  |
| (1) Trouble at the time of the experiment | 6.55 | 2.85 |  | 3.06 | 2.69 |  | *t*(31.87) = 3.67, *p* < .001, *d* = 1.25 |
| (2) Trouble in its worst… | 8.55 | 1.54 |  | 8.31 | 1.66 |  | *p*_wilcox_ = .71, *r* = .06 |
| (3) Goal Importance | 8.22 | 1.80 |  | 7.81 | 2.29 |  | *p*_wilcox_ = .68, *r* = .07 |
| (4) Example for general problems | 5.67 | 2.30 |  | 5.94 | 1.84 |  | *t*(31.69) = -0.38, *p* = .71, *d* = 0.13 |
| (5) Duration of the problem | 4.33 | 2.14 |  | 4.81 | 2.48 |  | *p*_wilcox_ = .46, *r* = .12 |
| (6) Time spent with the problem | 6.44 | 2.25 |  | 5.06 | 2.74 |  | *t*(29.15) = 1.59, *p* = .12, *d* = 0.55 |
| ***Goal evaluation*** |  |  |  |  |  |  |  |
| (1) Focus on negative aspects | 3.31 | 1.38 |  | 2.62 | 0.96 |  | *t*(31.96) = 1.74, *p* = .09, *d* = 0.57 |
| (2) Athlete felt worse | 3.05 | 0.85 |  | 3.06 | 0.93 |  | *p*_wilcox_ = .96, *r* < .01 |
| (3) Problem seemed worse | 2.95 | 0.97 |  | 2.06 | 0.77 |  | *p*_wilcox_ < .01, *r* = .47 |
| (4) Focus on bad feelings | 2.89 | 0.94 |  | 2.06 | 1.06 |  | *p*_wilcox_ < .01, *r* = .44 |

*Note.* EC = Experimental condition, GRCC = Goal-related control condition, *d* = Cohens *d,* *r* = respective effect size for Wilcoxon signed-rank test.* due to technical problems, only 18 athletes from the EC responded.

## **Performance in the ball juggling test**

Table S2 shows means and standard deviations of the scores obtained for the first and second trials of the performance test, as well as the means and standard deviations for the maximum score obtained for each of the two trials and their sum score.

**Table S2**

*Means (M), standard deviations (SD), and 95% confidence interval (CI) of the achieved points in the ball juggling test separated by time (performance test 1 vs. performance test 2) and condition.*

|  |  |  | |  | | **Performance of ball juggling** | | |  |
| --- | --- | --- | --- | --- | --- | --- | --- | --- | --- |
|  | First Trial |  | Second Trial |  | Max score | |  | Sum score |  |
| **Performance test 1** | *M* (*SD*) | *95%CI* | *M* (*SD*) | *95%CI* | *M* (*SD*) | | *95%CI* | *M* (*SD*) | *95%CI* |
| Experimental condition | 7.74 (5.61) | [5.03, 10.44] | 6.47 (6.38) | [3.40, 9.54] | 10.16 (6.14) | | [7.20, 13.12] | 14.21 (9.45) | [9.66, 18.76] |
| Goal-related control condition | 5.87 (4.54) | [3.45, 8.30] | 6.94 (5.35) | [4.09, 9.79] | 7.87 (5.34) | | [5.03, 10.72] | 12.81 (9.11) | [7.96, 17.67] |
| Neutral control condition | 6.20 (5.11) | [3.37, 9.03] | 4.00 (3.60) | [2.00, 6.00] | 6.73 (4.83) | | [4.06, 9.41] | 10.20 (7.90) | [5.82, 14.58] |
| **Performance test 2** |  |  |  |  |  | |  |  |  |
| Experimental condition | 6.53 (4.69) | [4.26, 8.79] | 6.63 (6.53) | [3.48, 9.78] | 8.36 (6.08) | | [5.43, 11.30] | 13.16 (9.83) | [8.42, 17.89] |
| Goal-related control condition | 7.69 (5.70) | [4.65, 10.72] | 4.87 (3.36) | [3.08, 6.67] | 8.56 (5.11) | | [5.84, 11.29] | 12.56 (7.92) | [8.34, 16.78] |
| Neutral control condition | 5.67 (5.29) | [2.74, 8.59] | 6.33 (5.26) | [3.42, 9.25] | 7.33 (5.26) | | [4.42, 10.25] | 12.00 (9.72) | [6.62, 17.38] |
